# Supplementary material for: Formation, collective motion, and merging of macroscopic bacterial aggregates
Source: PLoS Comput Biol. 2022 Jan 4;18(1):e1009153. doi: 10.1371/journal.pcbi.1009153 (PMC8759663; doi:10.1371/journal.pcbi.1009153)
Supplement: S2 Appendix — (PDF) [file pcbi.1009153.s008.pdf]

Simulation Equations

The parameters for the simulation are provided in the SI but also added in this document for the convenience of the reader. The components of the simulation have been adapted from (1).

Three states of motility

1. Chemotactic drift with random walk:

$$\frac{d\mathbf{r}_i}{dt} = \frac{1}{\gamma}\mathbf{F}_{chem}(\mathbf{r}_i, t) + \sqrt{2D}\boldsymbol{\xi}_i(t) \quad (1)$$

Where $\boldsymbol{r}_i$ is the two-dimensional position vector of a single agent, γ is the friction term (params_friction_gamma0 in the parameter table) and $\boldsymbol{F}_{chem}$ is the chemotactic drift force specified in detail in Eq 6 below. The second term models the random movement of the agents, with noise intensity D (params_noiseAmp /params_friction_gamma^2 in the parameter table), introduced with the Gaussian white noise vector $\boldsymbol{\xi}_i(t)$.

2. Immotile state:

$$\frac{d\mathbf{r}_i}{dt} = 0 \quad (2)$$

3. Random walk state:

$$\frac{d\mathbf{r}_i}{dt} = \sqrt{2D}\boldsymbol{\xi}_i(t) \quad (3)$$

Finite particle size

Eqns from 1-3 all included a short-range spring force term to simulate finite size.

$$\mathbf{F}_{spring}(\mathbf{r}_i, t) = \frac{k}{\gamma}\sum_{nn}(2r_s - d_{nn})\hat{\mathbf{n}}_{nn} \quad (4)$$

Where γ is the friction term, k is the spring constant (params_SpringConst in the parameter table). “nn” denotes nearest neighbors that are within two times the particle radius r_s (set by params_particleRadius in the parameter table). d_{nn} is the distance between the target nearest neighbor agent in question and the particle i. Finally, $\hat{\boldsymbol{n}}_{nn}$ denotes the unit vector pointing away from the direction of the nearest neighbor in relation to particle i.

Chemotactic drift term :

$$\mathbf{F}_{chem}(\mathbf{r}_i, t) = \frac{\kappa}{(1 + \beta c(\mathbf{r}_i, t))^2} \nabla c(\mathbf{r}_i, t) \qquad (5)$$

In the term above, κ (corresponding to params_ChemForce_kappa in the parameter table), modulates the strength of the chemotactic force. Furthermore, the parameter β sets the chemotactic saturation magnitude (params_ChemForce_beta). For more details and motivation on this chemotactic response see (1).

Chemotactic ligand production diffusion and decay term:

$$\frac{\partial c(\mathbf{r}, t)}{\partial t} = q_c \sum_{i=1}^{N} \delta(\mathbf{r} - \mathbf{r}_i(t)) - d_c c(\mathbf{r}, t) + D_c \Delta c(\mathbf{r}, t) \qquad (6)$$

This implementation is an extension of the original simulation taken from (1) . In essence, the original simulation utilized equations 1, 5 and 6 (without the spring force for finite particle size). In this work, an additional chemotactic concentration dependent motility transition was introduced, to mimic the experimental observations. The implementation is explained in detail in the following section.

Implementation of chemoattractant dependent motility switch

For each cell, a timer it $\boldsymbol{\tau}_i$ is assigned and initialized at the value of zero.

If we have the mathematical condition bellow, corresponding to high chemoattractant concentration (and thus a crowded position):

$$c(\mathbf{r}_i, t) > c_{threshold}$$

*Note that the $c_{threshold}$ is params_sitter_threshold in the parameter table and $c(\boldsymbol{r}_i, t)$ is the chemoattractant concentration at the position of the ith agent at time t.

Then the cell becomes immotile, (Eq 2), the timer starts, counting the time that the agent is in the immotile state. When the timer exceeds a threshold, set by params_t_escape in the parameter table, then the cell can stochastically regain its motility. The probability at each iteration for a cell to regain motility is given by params_p_escape in the parameter table. After the cell regains motility, it has a refractory period set by params_t_random_walk in the parameter table, in which it performs random motion without the influence of chemoattractant force given by Eq 3. After the time is elapsed, the cell reverts to the motion described in Eq 1. This is realized in the code by setting the timer, τ_i of the cell equal to a negative value equal to the negative of params_t_random_walk. At each iteration, the value diminishes according to the time elapsed in the random walk state, until it reaches to 0, where it pauses, and the cell obeys Eq 1.

At any time during this process if the chemoattractant drops below the threshold:

$$c(\mathbf{r}_i, t) \leq c_{threshold}$$

Then the cell regains its motility and reverts to the motion seen by Eq 1 (unless the cell is already in the random walk state it will remain until the time is elapsed).

This concludes the interactions set in the simulations. Note that the simulations, unless specified otherwise, were run for 100,000 agents in a 384x384 pixel square region with periodic boundary conditions.

1. Meyer M, Schimansky-Geier L, Romanczuk P. Active Brownian agents with concentration-dependent chemotactic sensitivity. Phys Rev E. 2014 Feb 13;89(2):022711.

Parameter Table:

Parameter name in file	Parameter raw magnitude
params_friction_gamma0	1
params_ChemForce_kappa	10
params_ChemForce_beta	1
params_noiseAmp	1
params_ChemField_prod	0.5
params_ChemField_decay	0.4
params_ChemField_diff	1
params_SpringConst	5
params_particleRadius	0.1
params_sitter_threshold	6
params_t_escape	1
params_t_random_walk	5
params_p_escape	0.0005
params_worldOrigin.x	0
params_worldOrigin.y	0
params_delta_t	0.005
params_simtime	2000
params_output	10
