# Supplementary material for: Formation, collective motion, and merging of macroscopic bacterial aggregates
Source: PLoS Comput Biol. 2022 Jan 4;18(1):e1009153. doi: 10.1371/journal.pcbi.1009153 (PMC8759663; doi:10.1371/journal.pcbi.1009153)
Supplement: S3 Appendix — (PDF) [file pcbi.1009153.s009.pdf]

A steady-state aggregate size for cells undergoing a motility transition

This model assumes that bacterial motility is a two-state system, namely a bacterium is either motile or immotile. The transition between these two states is governed by the bacterial activity. Experiments have shown that the immotile state is favored at high bacterial density. Two ways of achieving a density dependent switch in behavior are to sense when an excreted molecule has exceeded a threshold concentration or to sense when an environmental factor, such as oxygen, has been depleted below a threshold. The model presented below does not depend on whether the cell is sensing the accumulation of an excreted molecule or depletion of an environmental factor. For concreteness we will assume accumulation of an excreted factor triggers the transition from the motile to immotile state.

As the population of cells grows over time, eventually the density will become high enough that a random fluctuation in local cell density cause some cells to transition to the immotile state. This "nucleation event" will create a region of immotile cells. Because these cells cannot move, the density fluctuation at that region is frozen in place. As motile cells swim into the region, they will also transition to the immotile state, and this positive feedback will cause the cluster of immotile cells to grow over time.

If the only microscopic rule in the system was the transition to an immotile state when cell density is high, this would result in all of the cells in the population accumulating into one or more clusters of immotile cells over time. In experiments, two observations suggest that the reverse transition, from the immotile to motile state, must be occurring even in regions with high cell density. First, over time, a population of motile cells is maintained. Second, at a microscopic level, the boundaries between regions of motile and immotile cells have been shown to recede over time. The recession of a boundary of immotile cells must be the result of cells at the edge transitioning back to motile state and swimming away. We assume that this transition from the immotile to motile state occurs randomly.

Therefore, the basic system consists of a motile cells that transition to the immotile state due to fluctuations in local cell density. Once a high-density region is nucleated, an aggregate of immotile cells forms. The aggregate grows as motile cells swim into the region and become immotile and the aggregate shrinks as cells regain motility and swim away. Would these microscopic rules lead to a characteristic and steady-state aggregate size?

A characteristic aggregate size would occur at an aggregate size when the rates of aggregate growth and reduction are matched. As the transition back to motility occurs randomly, the rate of cells leaving the aggregate would be proportional to the number of cells in the aggregate. Because aggregate area is proportional the number of cells in the aggregate, the rate of cells leaving the aggregate is directly proportional to aggregate area.

$$rate_{leaving} = \gamma A, \qquad \text{(Eq S2\_1)}$$

where γ is a rate constant that accounts for the rate of cells leaving the aggregate, A is the aggregate area in units of number of cells.

For a cell to join the aggregate and become immotile, a free, motile cell needs to run into an aggregate. Therefore, the rate of cells joining a given aggregate is proportional to the number free cells per aggregate times the aggregate area, as cells joining the aggregate requires a free cell to swim into an existing aggregate.

$$rate_{joining} = k(\frac{C_{free}}{n}A), \quad \text{(Eq S2\_2)}$$

where k is the rate constant of cells joining an aggregate, C_{free} is the total number of motile cells in the system, and n is the number of aggregates. Here we assume free cells randomly explore space and are not attracted or repelled to regions of high cell density. Any chemotactic movement could potentially be incorporated into the rate constant k to account for a reduction or increase in the rate of free cells joining an existing aggregate.

Free cells can be calculated by subtracting the number of cells in aggregates from the total number of cells in the system (),

$$C_{free} = C_{total} - A\,n. \quad \text{(Eq S2\_3)}$$

Combining these equations, we arrive at the rate of joining an aggregate to be,

$$rate_{joining} = k\left(\frac{C_{total} - A\,n}{n}A\right) = \frac{kC_{total}A}{n} - kA^2. \quad \text{(Eq S2\_4)}$$

As shown in Figure 1, plotting the rates of cells leaving and joining an aggregate versus aggregate area shows the potential of a stable, steady-state aggregate area. This plot includes a dashed line to indicate a minimal area, as below a critical size am aggregate would not reach the required local density to initiate the transition to the immotile state.

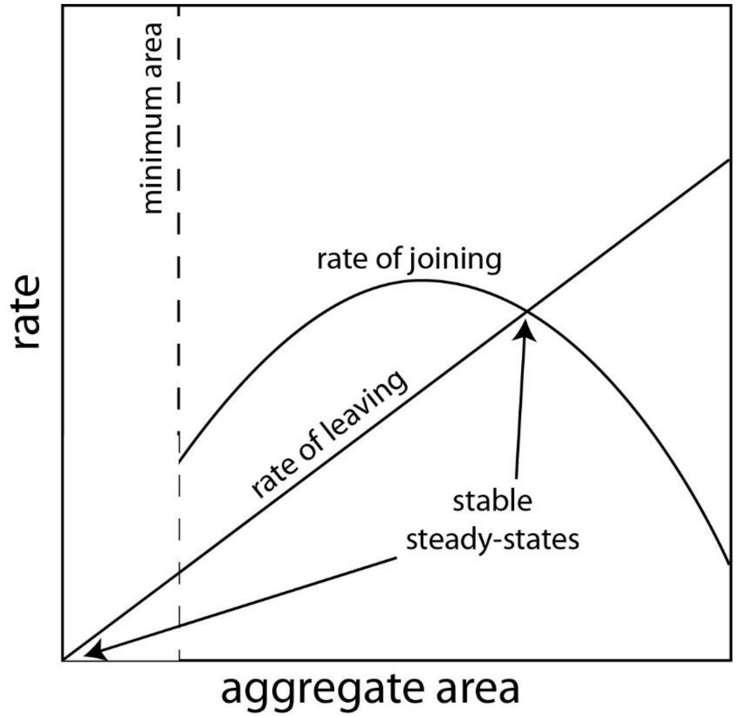

Figure SI 2_1: Competition between the rates of cells leaving and joining aggregates creates a characteristic aggregate area.

The steady-state area could be calculate by setting the rates of joining and leaving an aggregate equal to each other.

$$rate_{joining} = rate_{leaving} \quad \text{(Eq S2\_5)}$$

$$\gamma\, A = \frac{kC_{total}A}{n} - kA^2 \quad \text{(Eq S2\_6)}$$

which simplified to

$$A_{steady-state} = \frac{C_{total}}{n} - \gamma/k \quad \text{(Eq S2\_7)}$$

$\frac{C_{total}}{n}$ is the ratio of total number of cells to the number of aggregates and γ/k is the ratio of the rate constants for cells leaving to cells joining an aggregate. As γ/k goes to zero, the number of cells within an aggregate becomes $\frac{C_{total}}{n}$, indicating that all the cells divide over the n aggregates in the system. As γ/k increases, the number of cells per aggregate is reduced up until the point that all the cells in the system remain motile.
