# Supplementary figures and images for: Formation, collective motion, and merging of macroscopic bacterial aggregates
Source: PLoS Comput Biol. 2022 Jan 4;18(1):e1009153. doi: 10.1371/journal.pcbi.1009153 (PMC8759663; doi:10.1371/journal.pcbi.1009153)

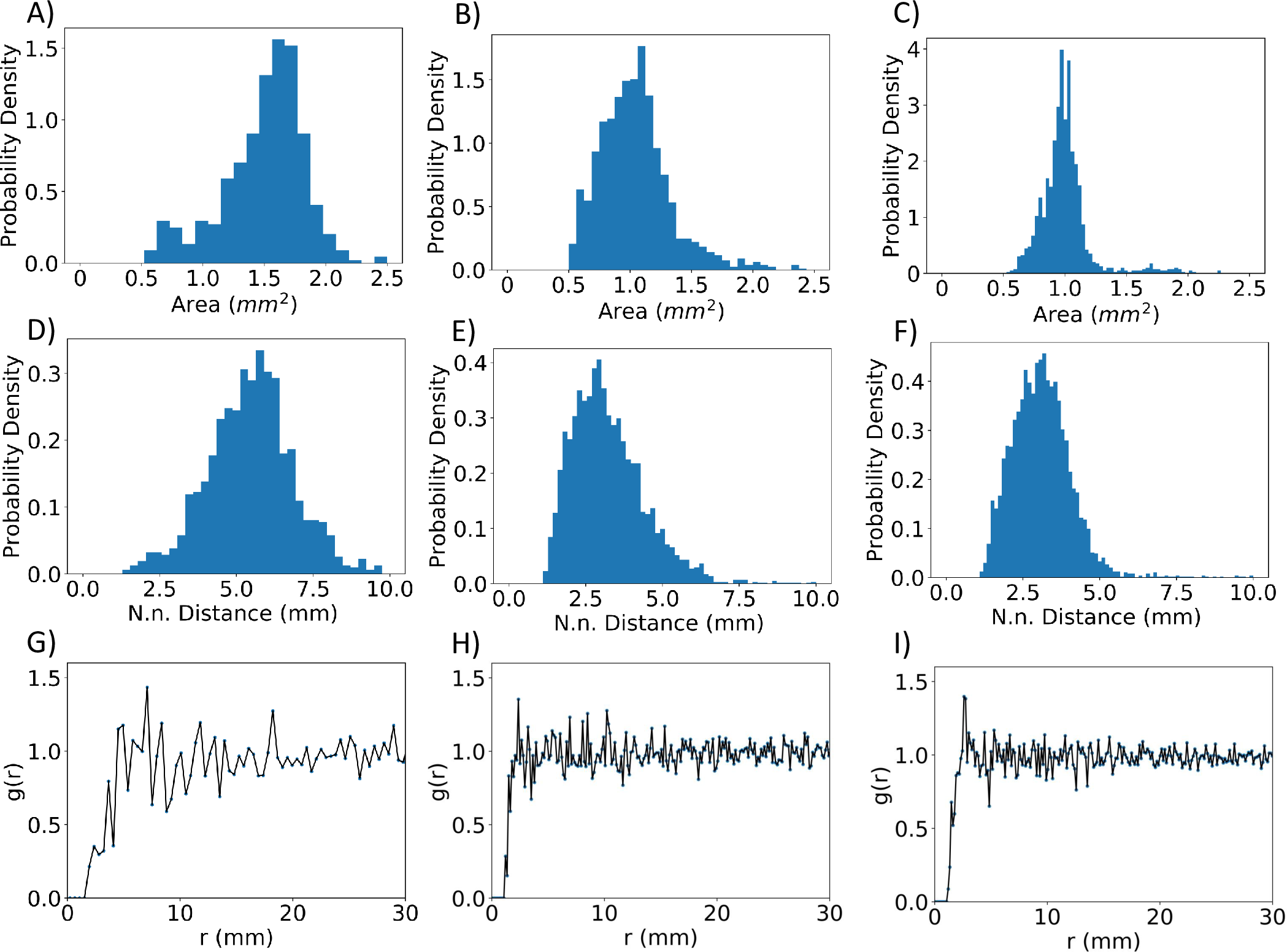

Supplement: S1 Fig — Spatial quantities are calculated for three different plates at 22.5 hrs. The first two columns are unused replicate experiments, and the third column is obtained from the plate used throughout the study. The quantities are calculated for a 10x10cm subregion in the center of the plate to account for edge effects. A-C) Aggregate area distributions. D-F) Nearest neighbor distance distributions. G-H) Pair correlation functions. (TIF) [file pcbi.1009153.s013.tif]

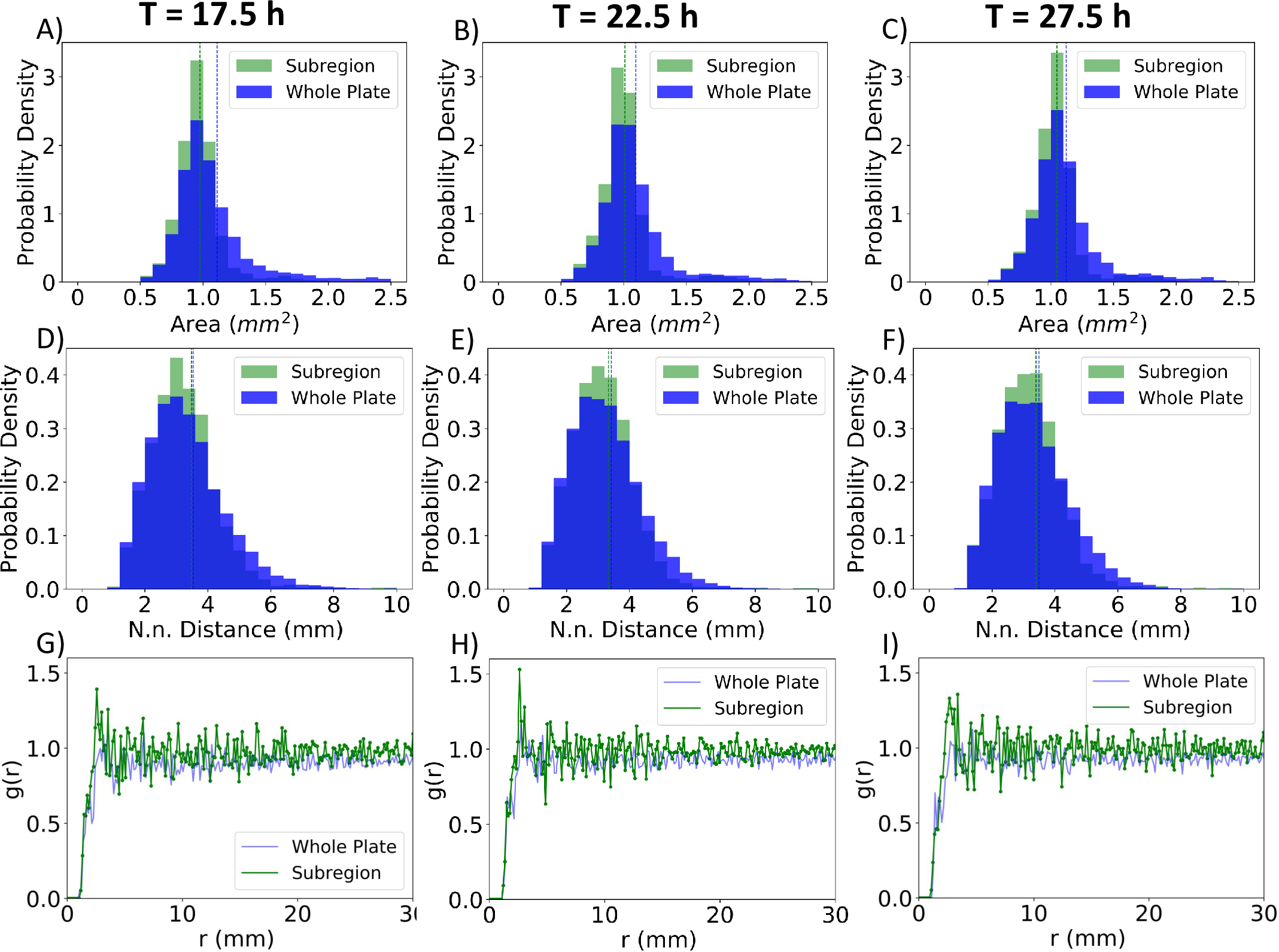

Supplement: S2 Fig — Spatial quantities for three time points 17.5 h, 22.5 h and 27.5 h from left to right. The quantities were plotted with blue for the whole plate and green for the subregion used in the main text, shown in S3 Fig. A-C) Aggregate area distributions. D-F) Nearest neighbor distance distributions. G-H) Pair correlation functions. (TIF) [file pcbi.1009153.s014.tif]

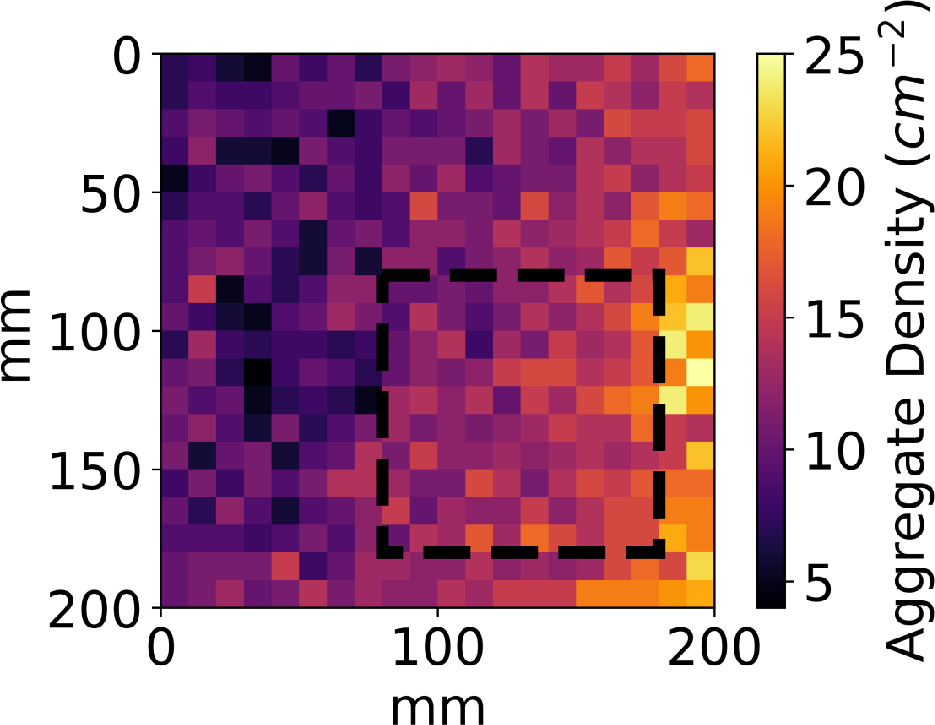

Supplement: S3 Fig — The aggregate number variations are visualized for the frame used for the spatial analysis, at T = 22.5 h. The highlighted region marks the spatial subset of the plate analyzed in Fig 2. (TIF) [file pcbi.1009153.s015.tif]

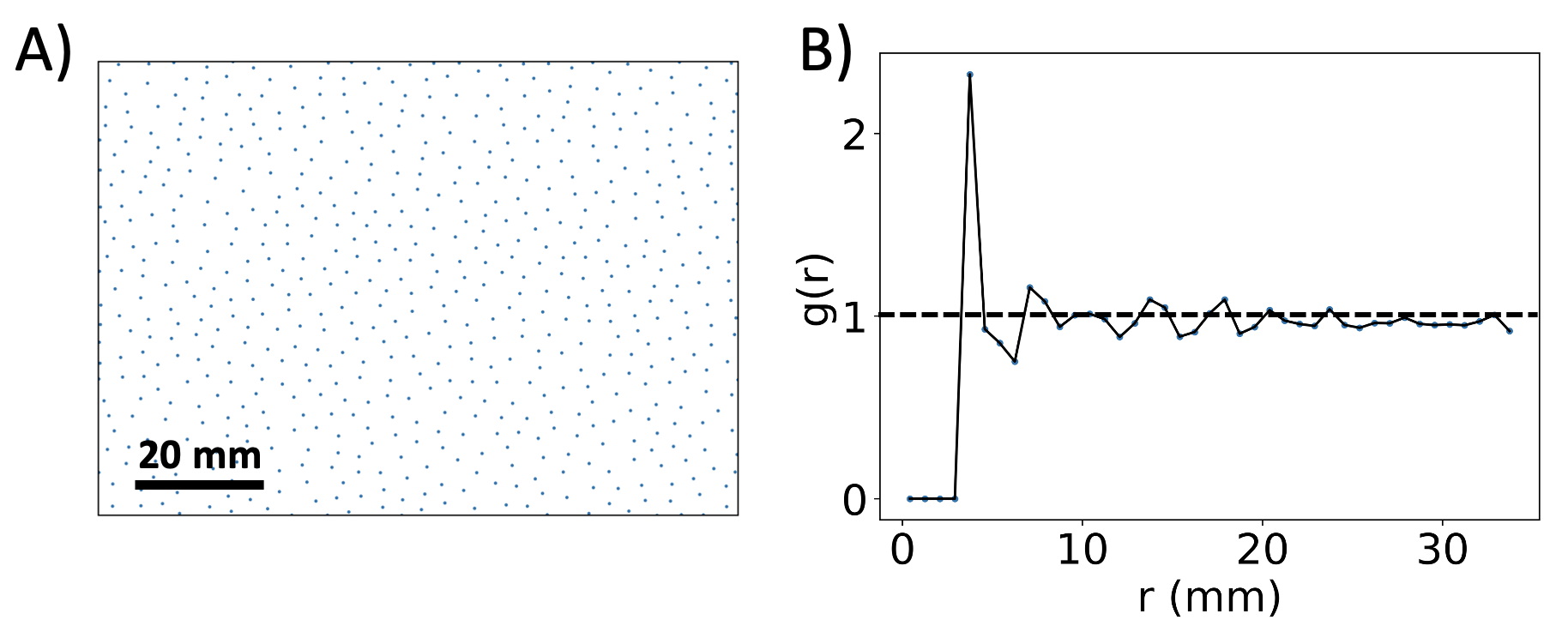

Supplement: S4 Fig — (A) A hard sphere model pattern in a 100x100m2 region generated by assigning 650 points a random position with the condition that they do not lie within a distance of 3.33 mm from any neighboring points. The value 3.33mm is taken from the average nearest neighbor distance in the experimental subregion analyzed in Fig 2. (B) Pair correlation of the respective point patterns. The dotted horizontal line represents the pair correlation factor of a random point pattern for large system size. The pair correlation of the hard sphere model captures the spatial characteristics observed in the pair correlation of the bacterial aggregate pattern. Note that a deterministic radius of exclusion makes the peak at the average nearest neighbor distance more pronounced than in the experiment and leads to a smoother curve. (TIF) [file pcbi.1009153.s016.tif]

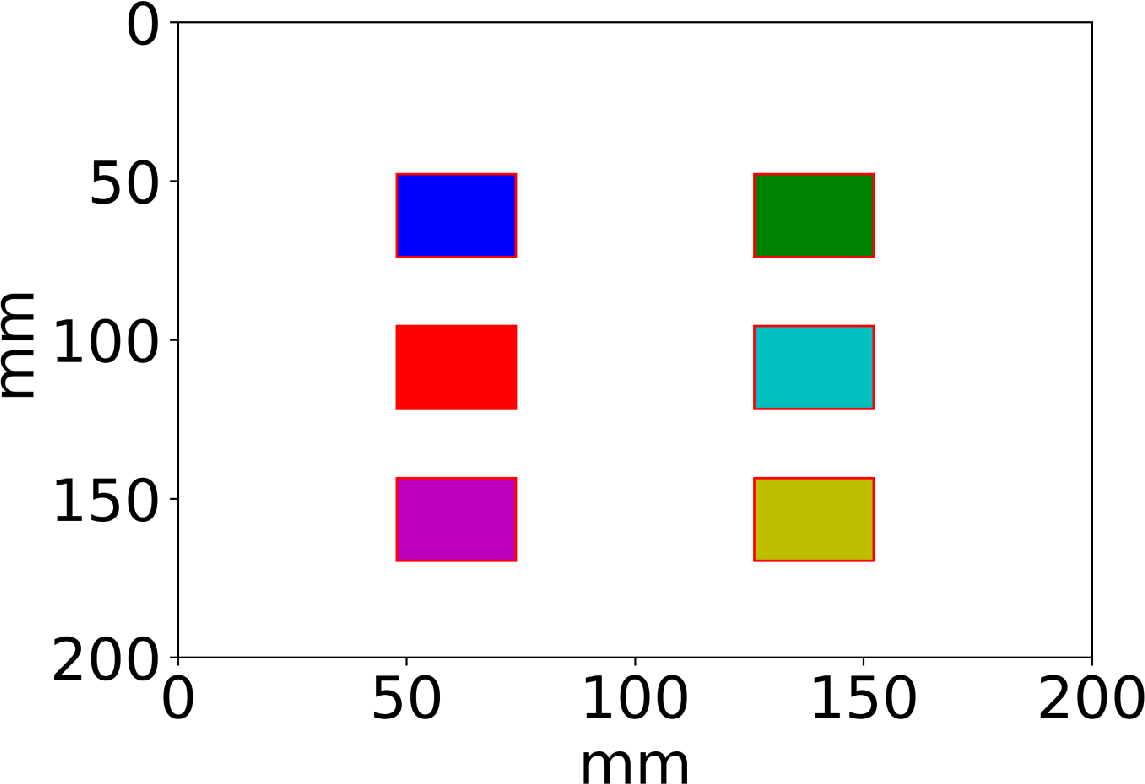

Supplement: S5 Fig — (TIF) [file pcbi.1009153.s017.tif]

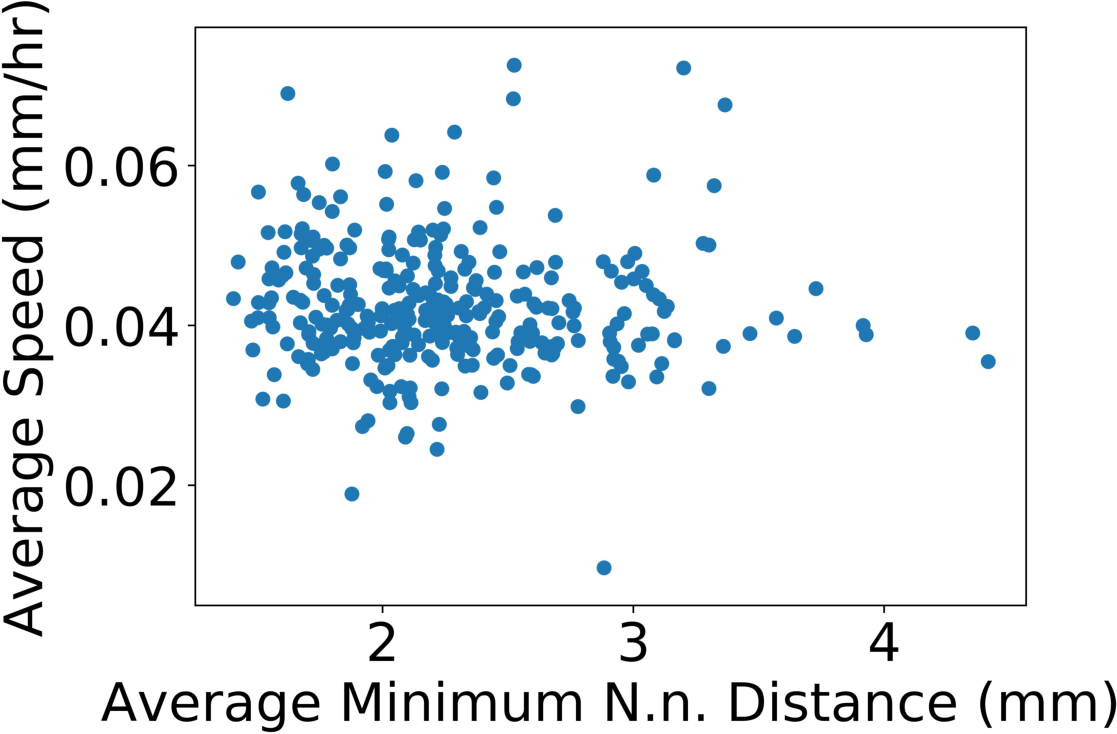

Supplement: S6 Fig — Average speed versus average minimum nearest neighbor distance for all analyzed aggregate trajectories. To obtain the latter quantity for a single trajectory, the nearest neighbor distances were calculated for each frame, the minimum was extracted and averaged over all frames. Spearman rank-order correlation coefficient correlation yielded -0.12, with a p-value of 0.034. (TIF) [file pcbi.1009153.s018.tif]

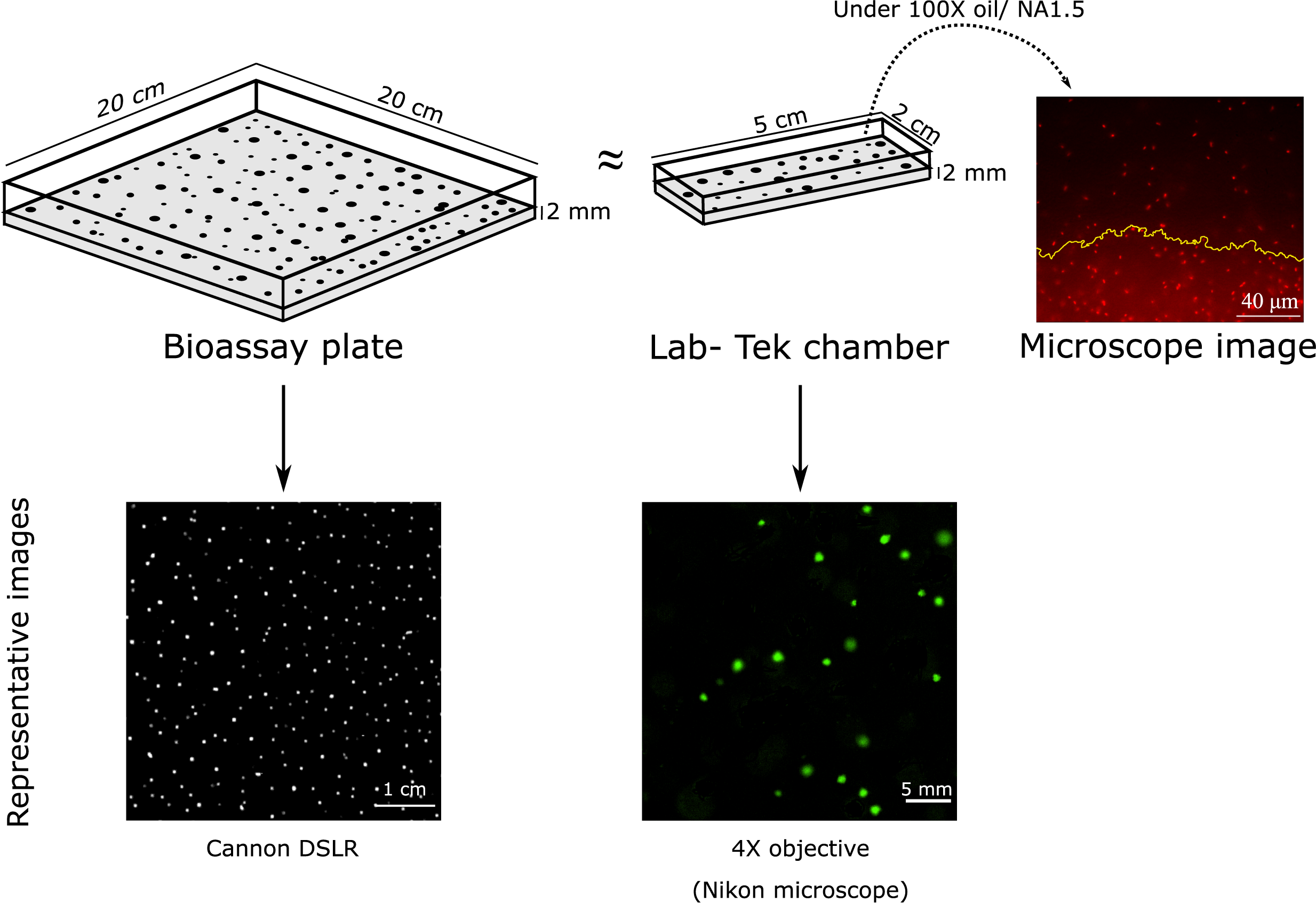

Supplement: S7 Fig — The height of agar in both the vessels was 2 mm. Representative images taken by using DLSR camera and 4X objective on Nikon microscope for respective set- up has been shown. Spot formation in the Lab-Tek chamber was imaged in time-lapse microscopy using 100X oil/1.5 NA objective. (TIF) [file pcbi.1009153.s019.tif]

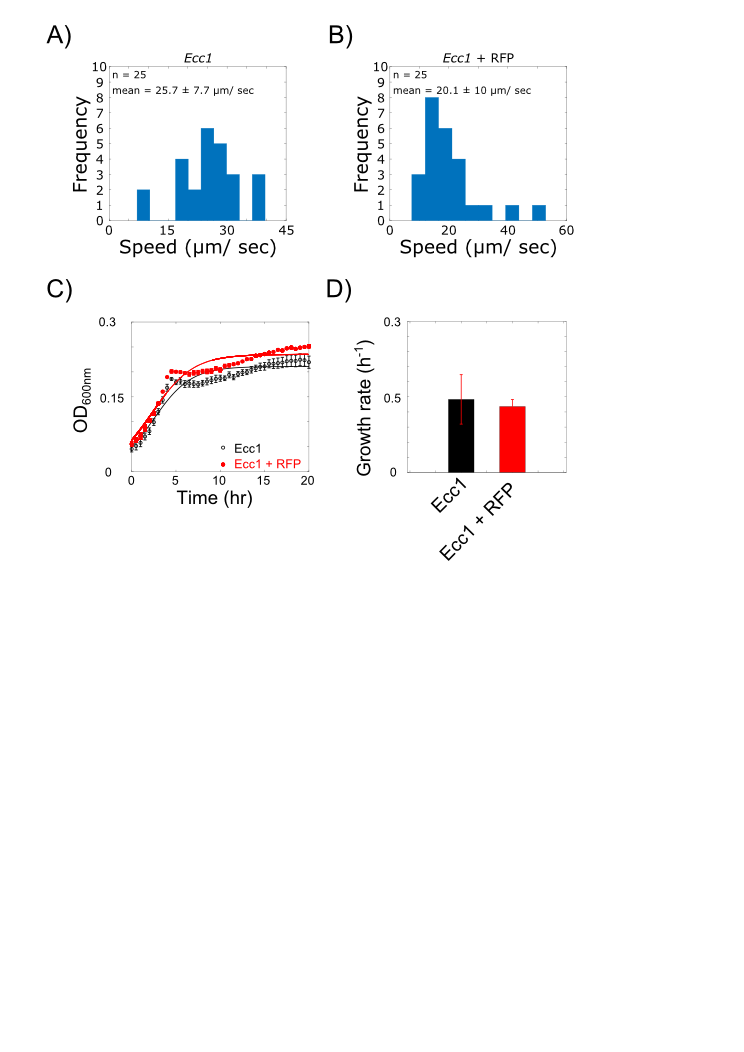

Supplement: S8 Fig — Enterobacter cloacae Ecc1 cells transformed to express RFP protein were compared with Enterobacter cloacae Ecc1 cells for single cell motility (A and B). (C) Their respective growth in M9 + Glucose at room temperature was fit to the logistic growth equation (solid lines) to calculate their respective growth rates as shown in (D). n = 3. Error bars- Standard deviations. (TIF) [file pcbi.1009153.s020.tif]

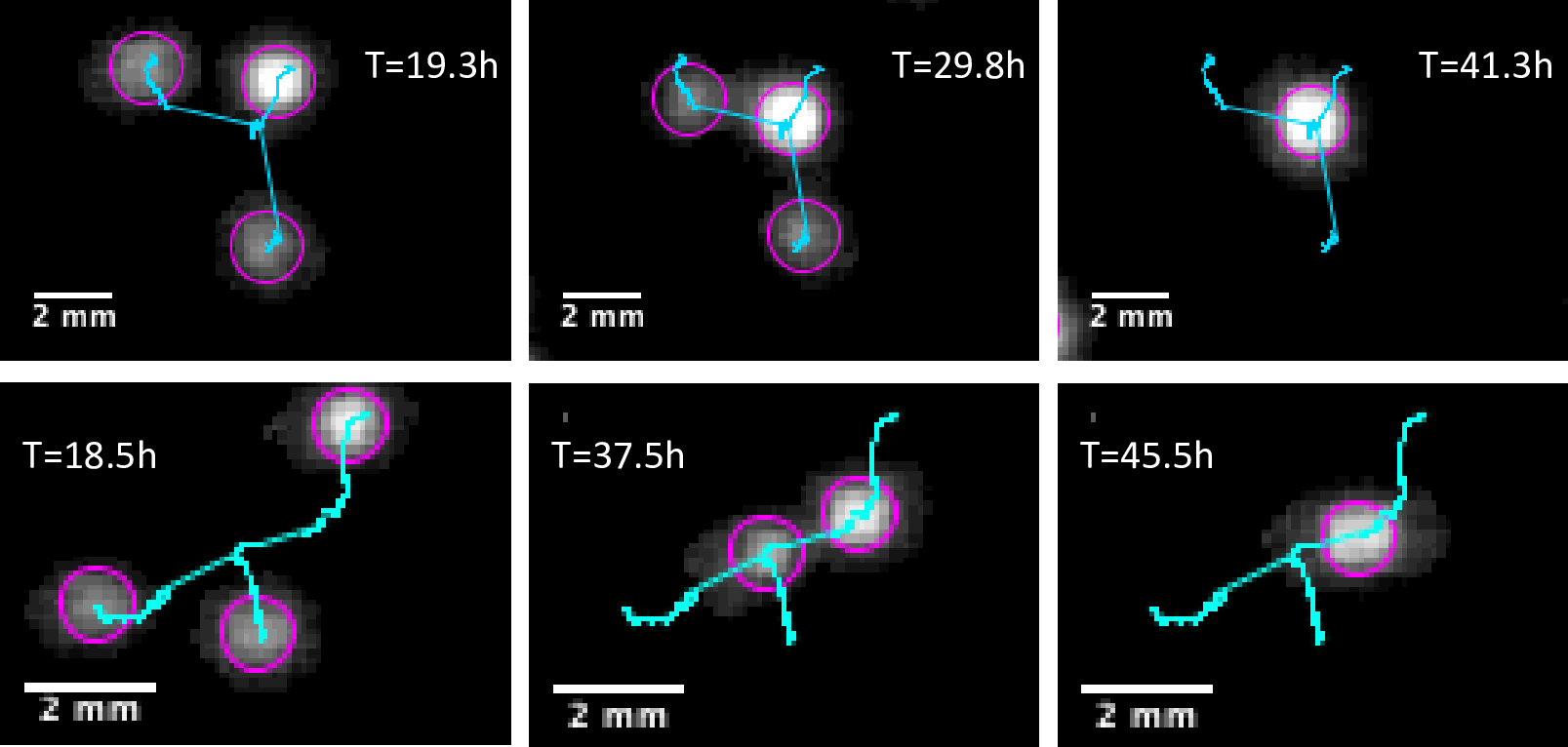

Supplement: S9 Fig — Merging of three aggregates. 4.2% of aggregates took part in a merger involving three aggregates. The top and bottom row show two instances of a three-aggregate merger over time. (TIF) [file pcbi.1009153.s021.tif]

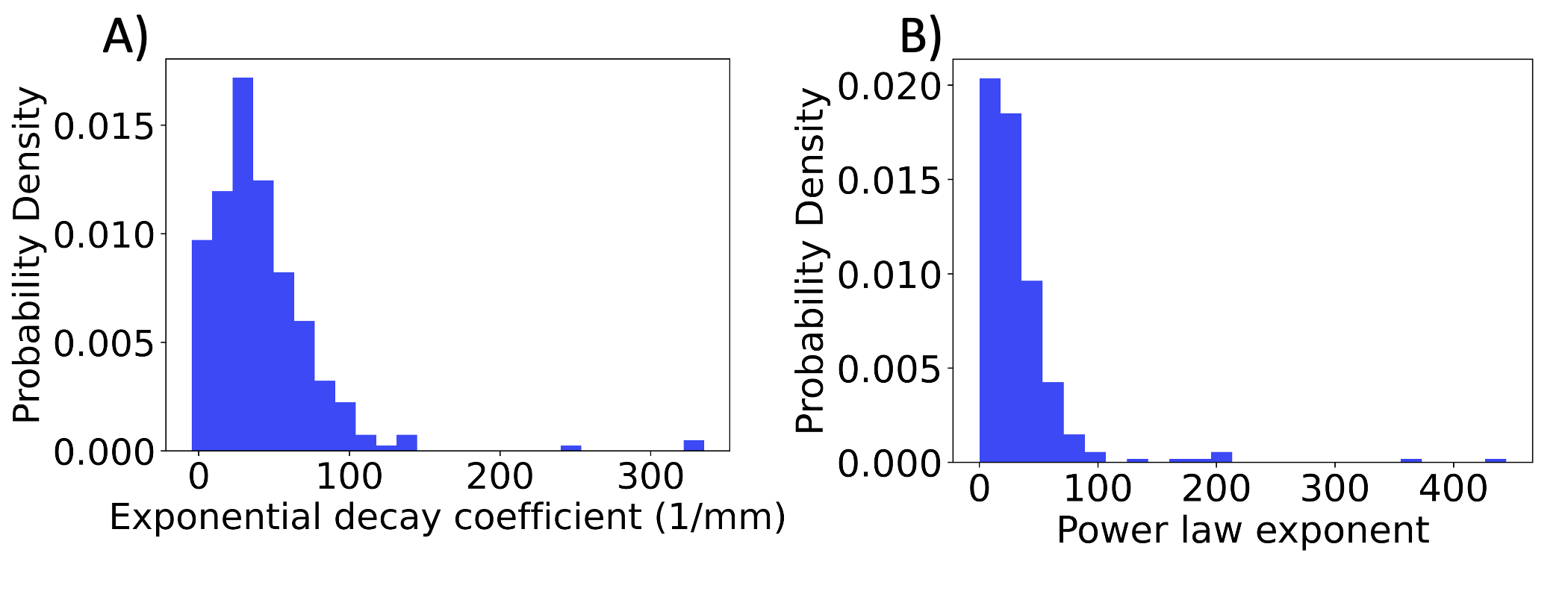

Supplement: S10 Fig — Probability density distributions of the coefficients obtained by fitting acceleration versus relative distance for each two-spot merging trajectory in Fig 3 with an exponential (A) and a power law (B). Exponents were included for fits that yielded exponents with one standard deviation error that was less than 50% of their respective value. (A) Distribution of the decay exponent, n, obtained by assuming an exponentially dependent force: a(drel)=A0e−ndrel. (B) Distribution of the power law exponent, n, obtained by assuming a power law dependent force: a(drel) = A0/dreln. It is not possible to distinguish whether a power or exponential law best describes the acceleration profile. Both distributions of the inferred coefficients are wide, a result that does not support the existence of an underlying exponential, or a power law dependent force. (TIF) [file pcbi.1009153.s022.tif]

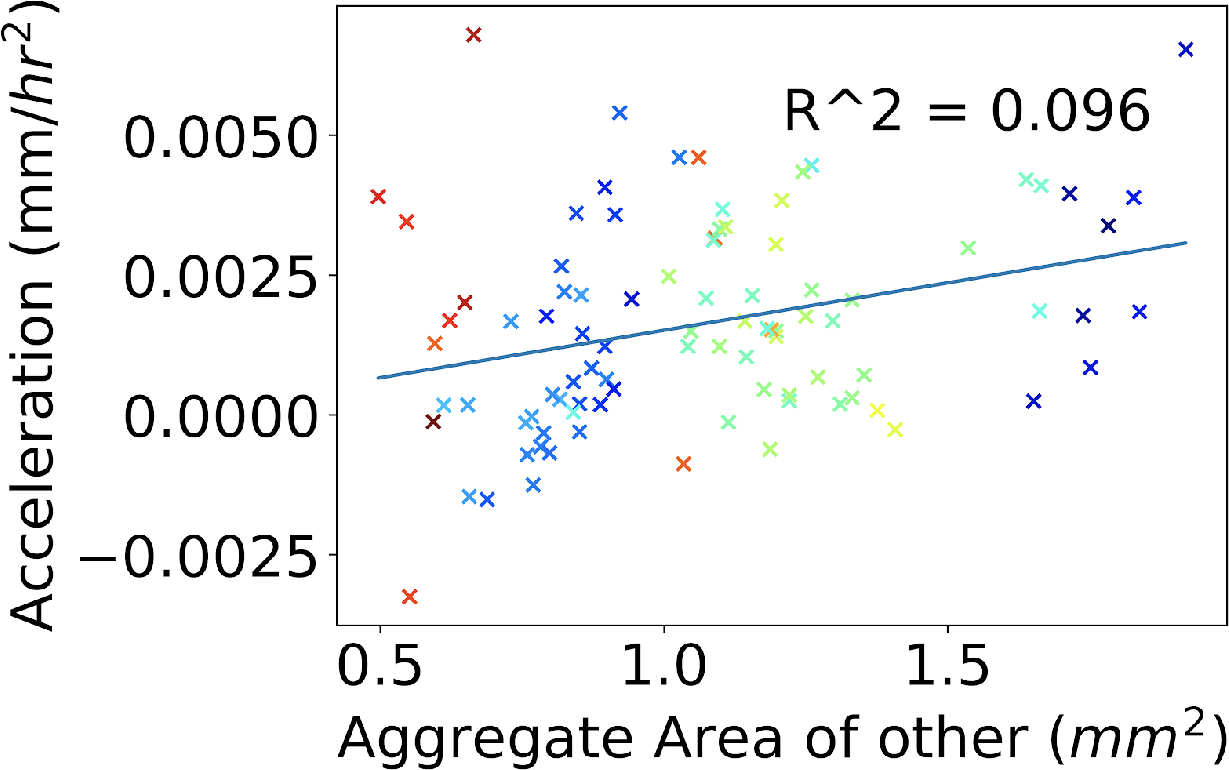

Supplement: S11 Fig — This is an additional analysis performed on two-spot merging trajectories observed in experimental measurements. In this instance, the distance of each spot relative to the collision point versus time is fitted to a quadratic equation. Approximating the trajectory as constant acceleration motion, the acceleration is obtained from the quadratic equation. Finally, it is plotted with respect to the other merging aggregate area. The two quantities have a spearman rank-order correlation coefficient of 0.31 with a p-value of 0.002. The color of each data point corresponds to a merging event taking place in the respectively colored subregion in S5 Fig. (TIF) [file pcbi.1009153.s023.tif]

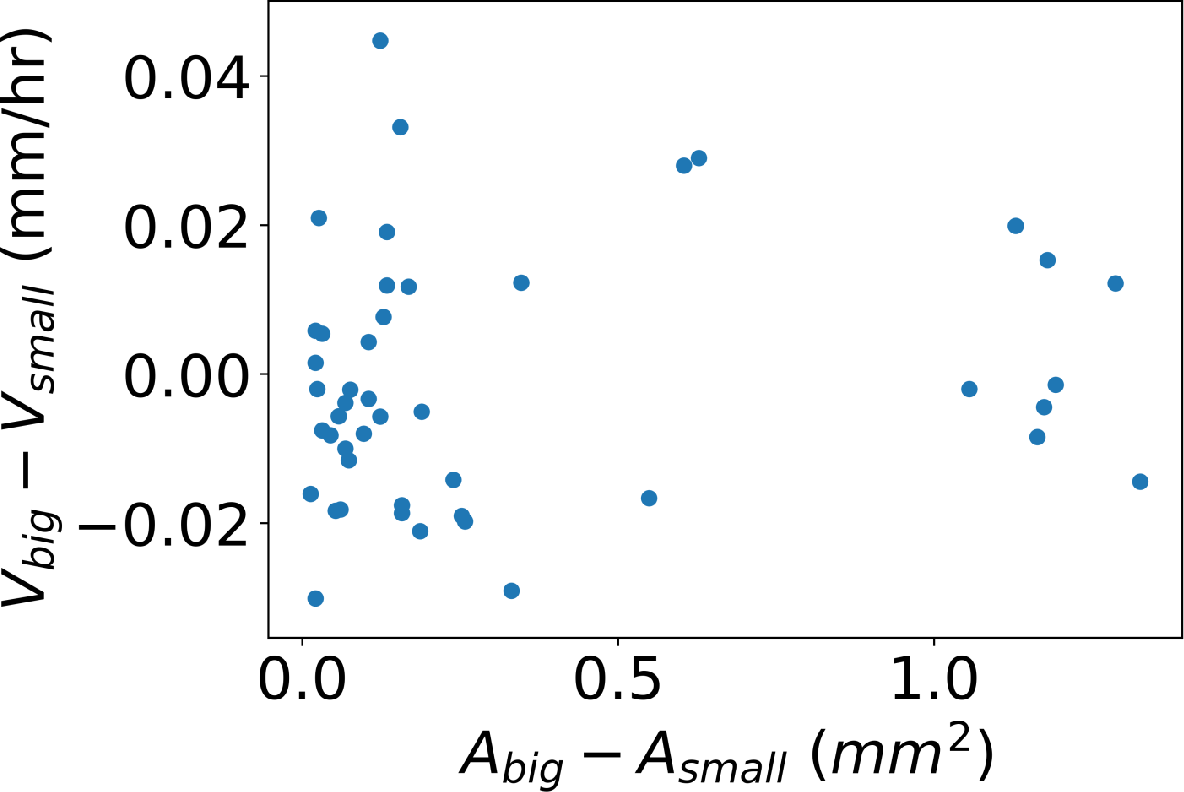

Supplement: S12 Fig — Small aggregates do not necessarily move faster than big aggregates during two-spot merging events. This is the case since the difference of speeds is not always positive, even when the size difference is significant. (TIF) [file pcbi.1009153.s024.tif]

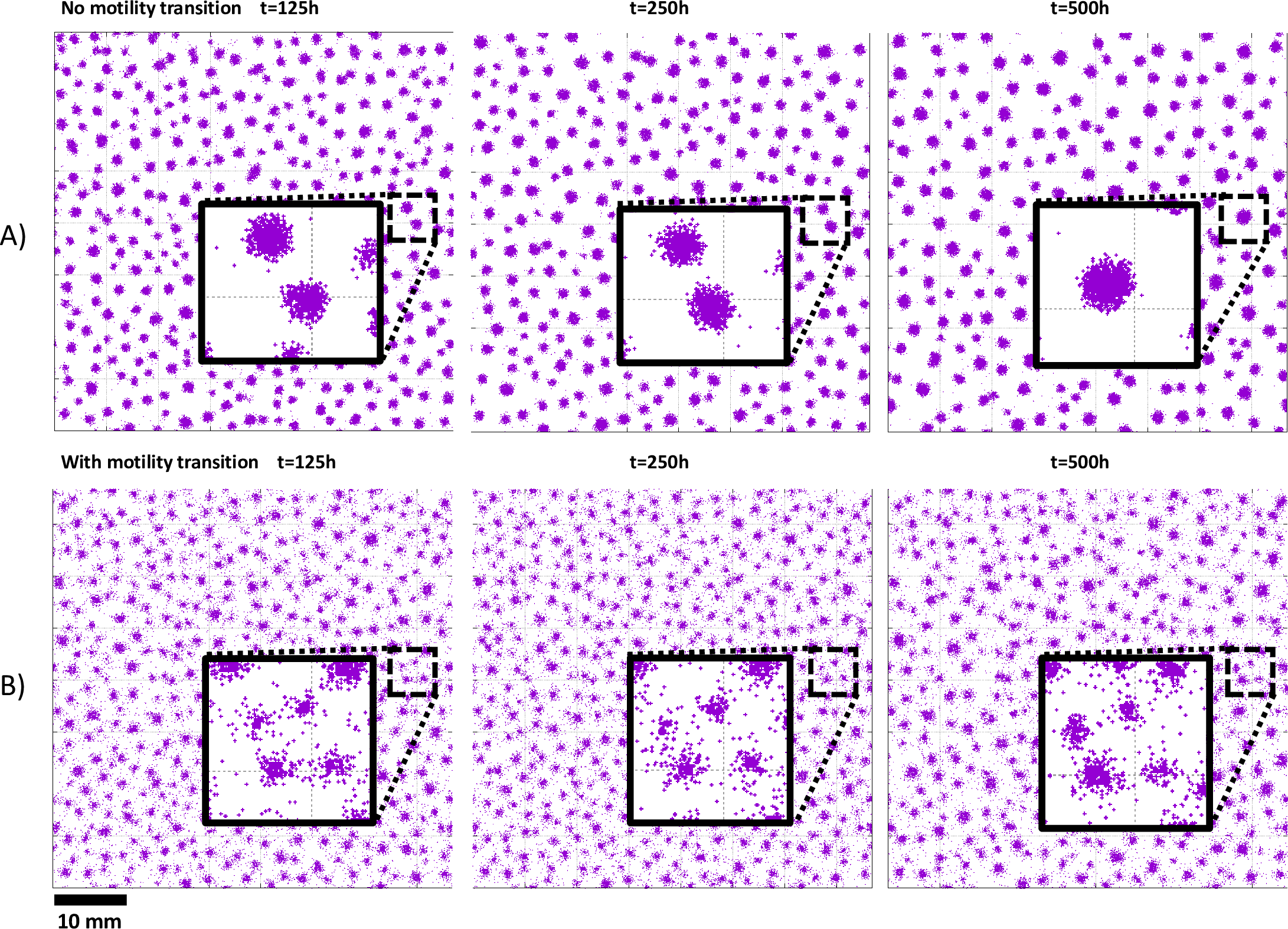

Supplement: S13 Fig — (A) With no motility transition. (B) With the motility transition. (TIF) [file pcbi.1009153.s025.tif]

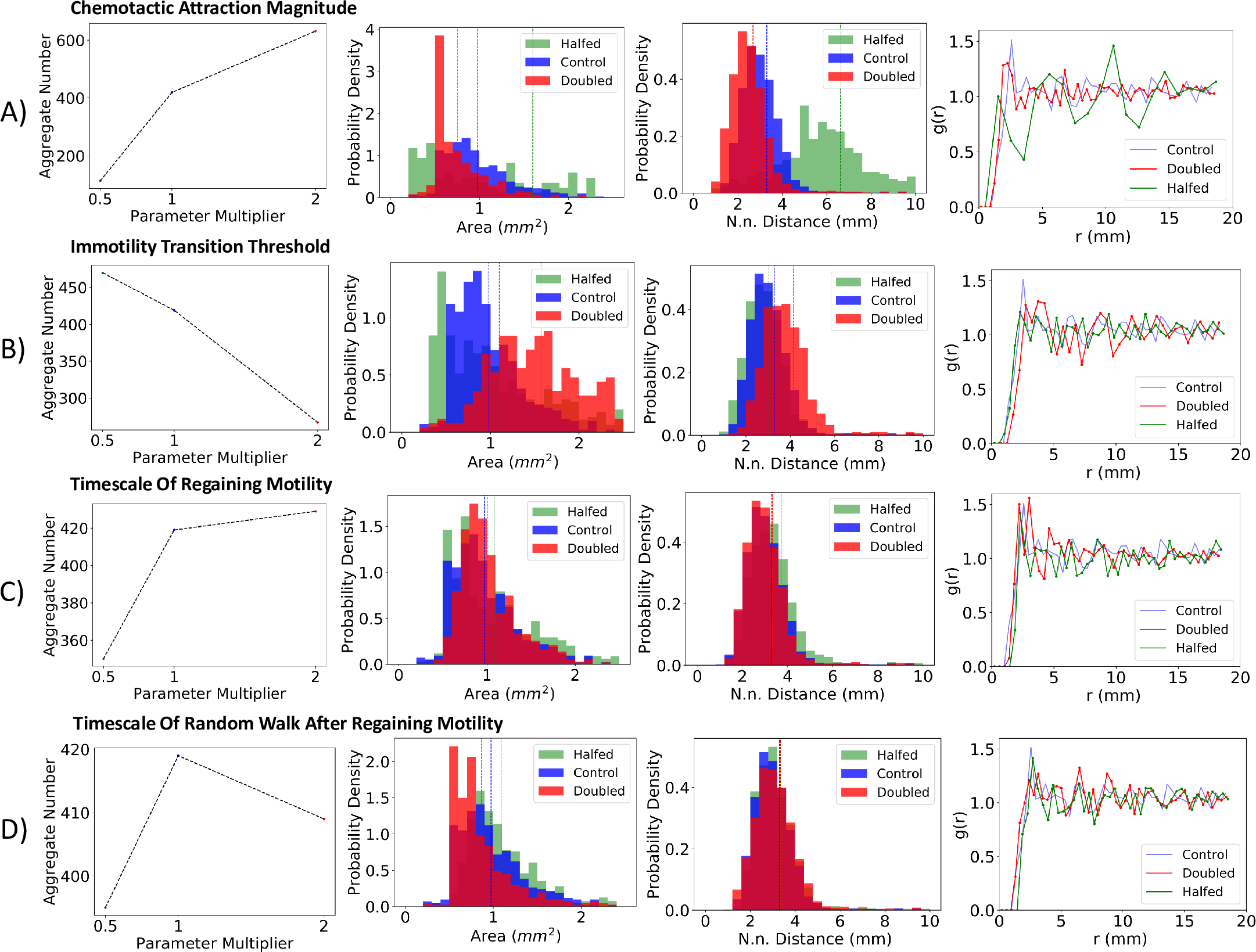

Supplement: S14 Fig — (A) Chemotactic attraction magnitude (B) Immotility transition threshold (C) Timescale of regaining motility (D) Timescale of random walk after regaining motility. (TIF) [file pcbi.1009153.s026.tif]

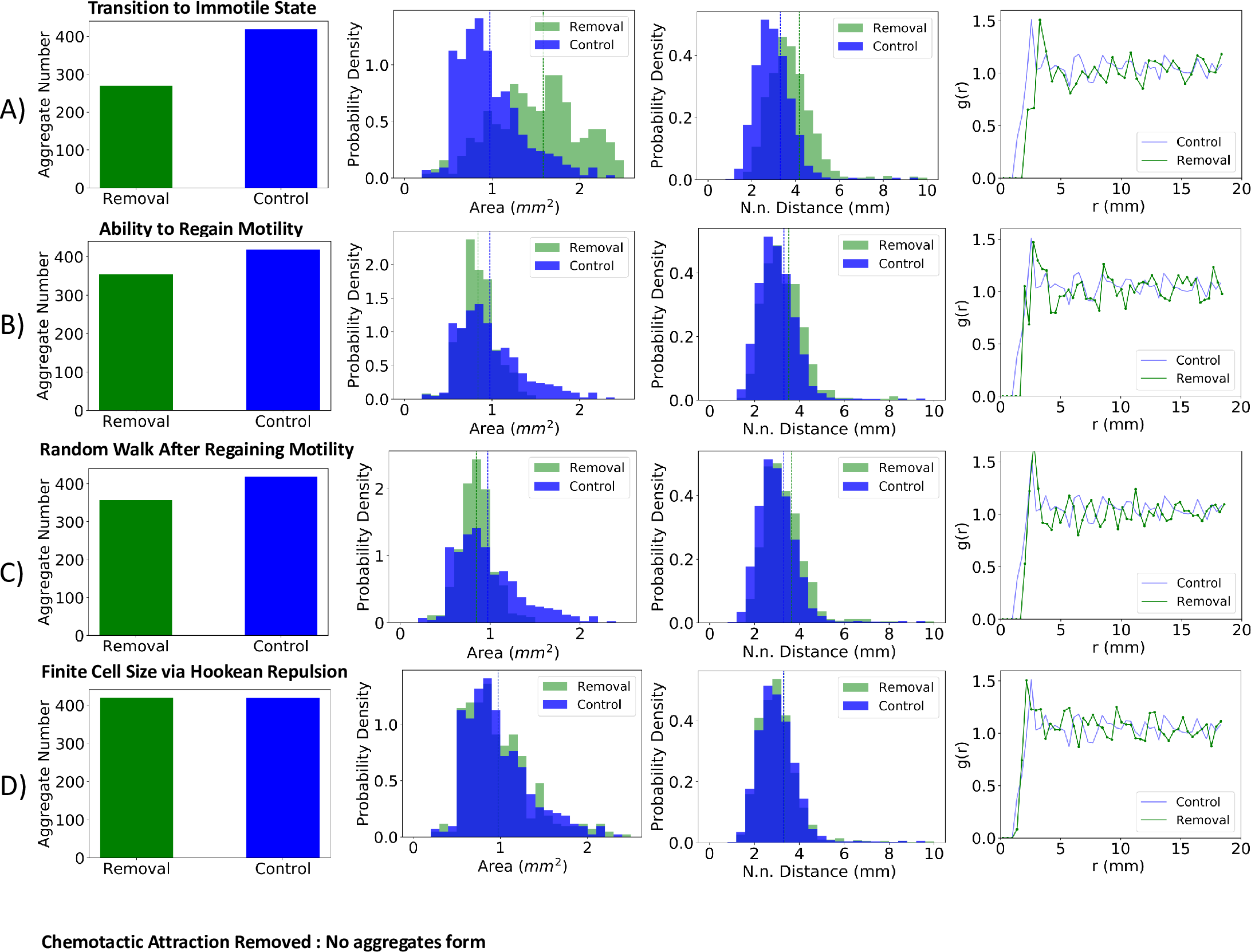

Supplement: S15 Fig — (A) Chemotactic attraction magnitude (B) Immotility transition threshold (C) Timescale of regaining motility (D) Timescale of random walk after regaining motility. (TIF) [file pcbi.1009153.s027.tif]

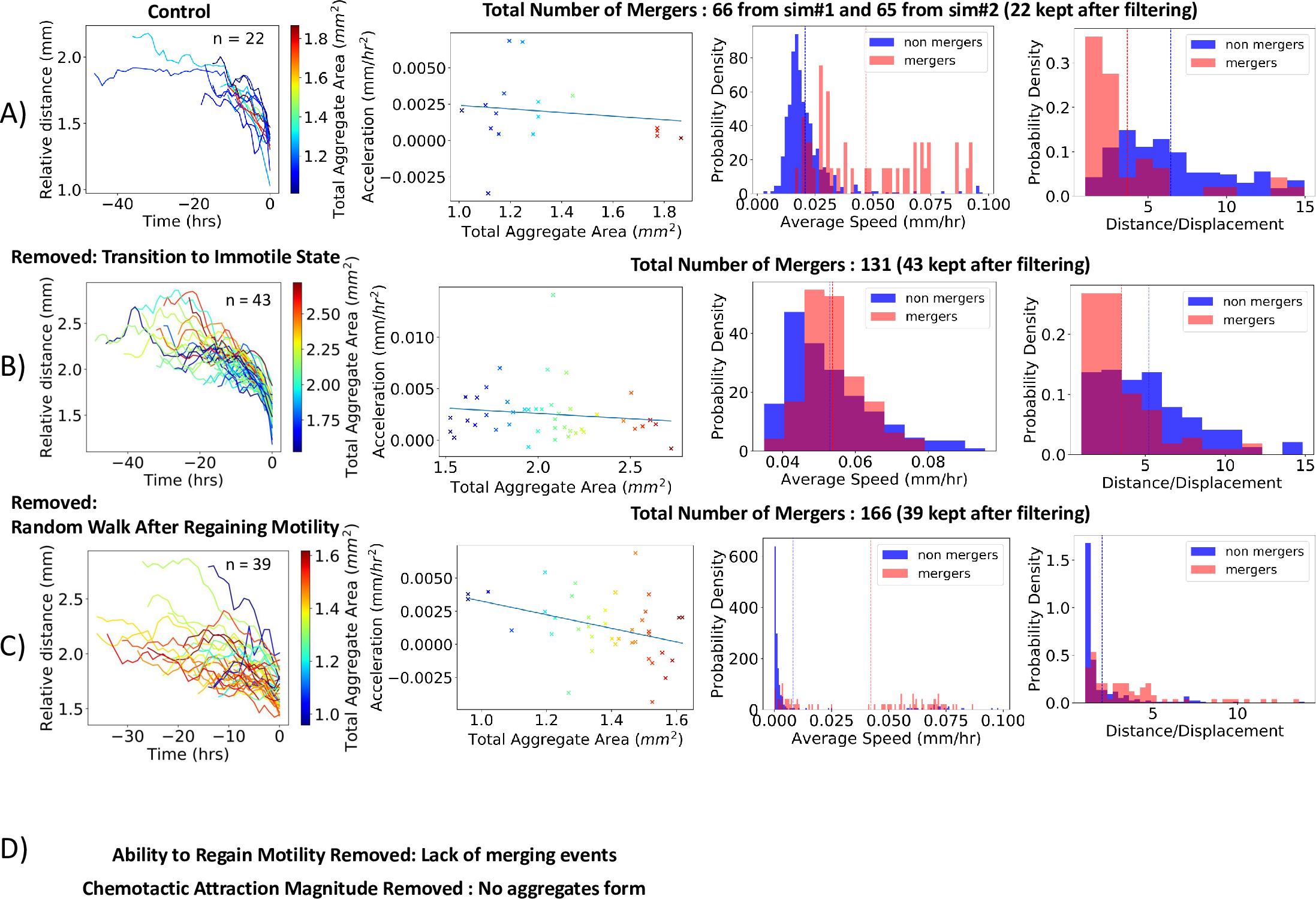

Supplement: S16 Fig — (A) Original simulation results for comparison (B) Removing the introduced motility transition (C) Removing random walk process after cells regain motility. (TIF) [file pcbi.1009153.s028.tif]

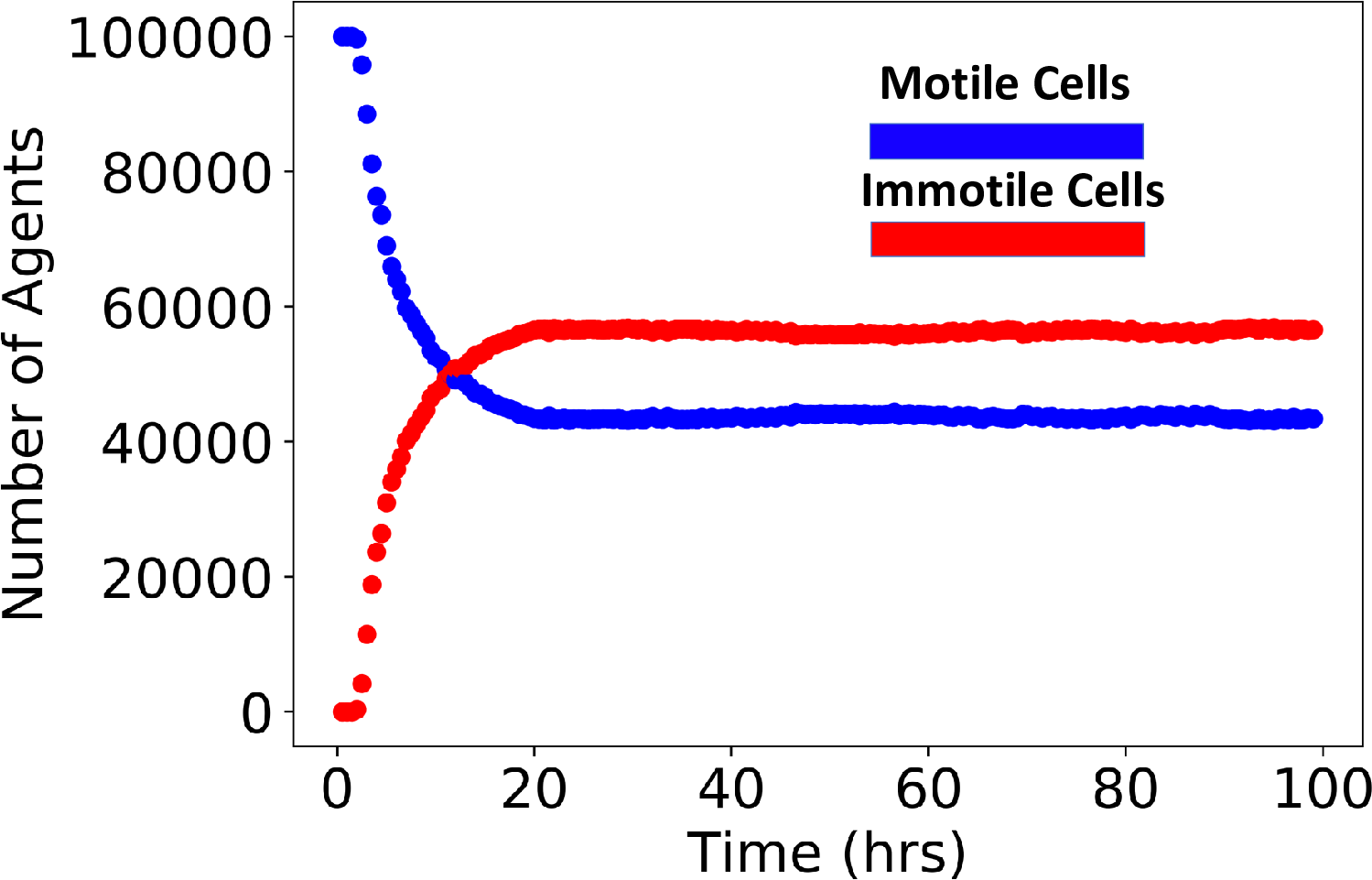

Supplement: S17 Fig — During the simulations, the distribution of motile and immotile cells reaches and retains a mixed steady state. (TIF) [file pcbi.1009153.s029.tif]

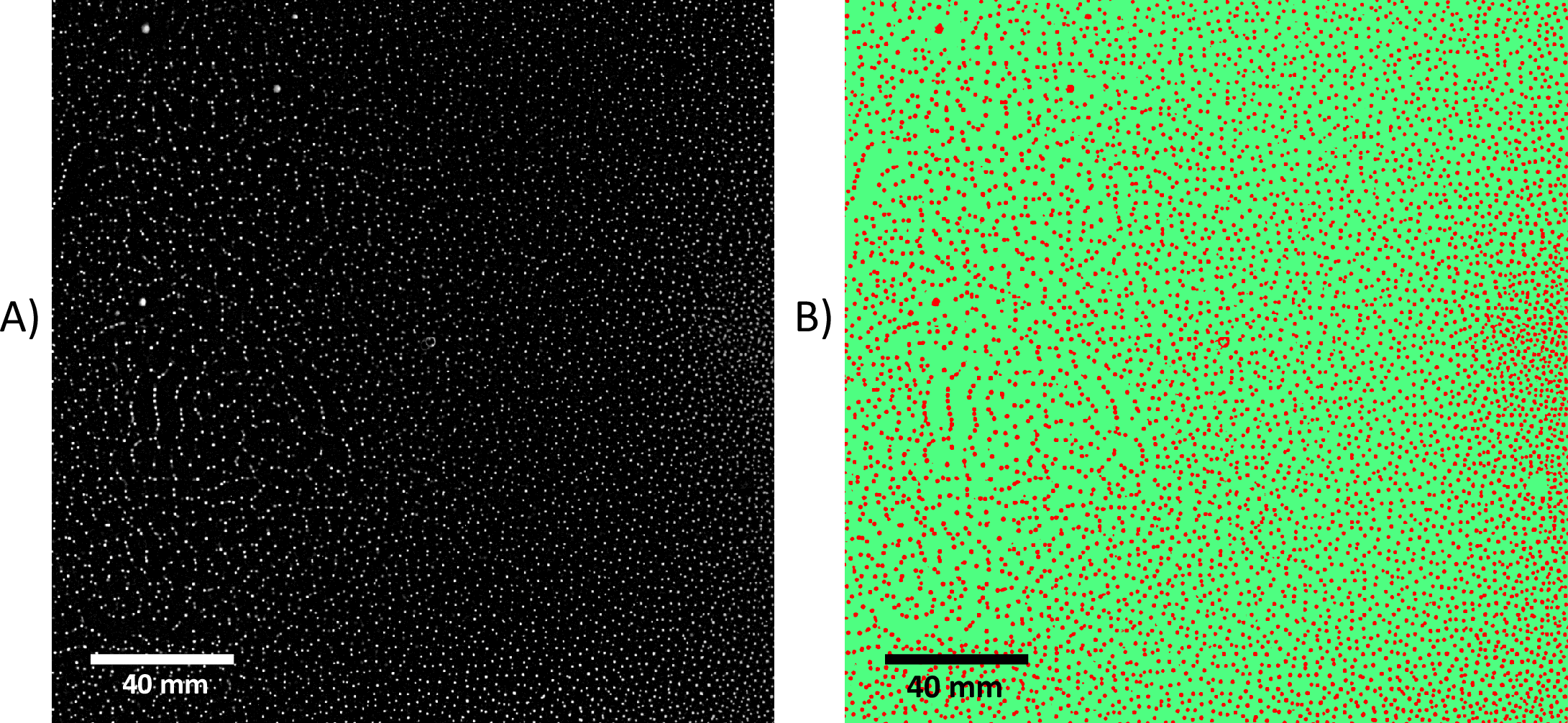

Supplement: S18 Fig — A) Experimental image of the entire plate at T = 22.5 h. B) Aggregates identified after using Weka software. (TIF) [file pcbi.1009153.s030.tif]

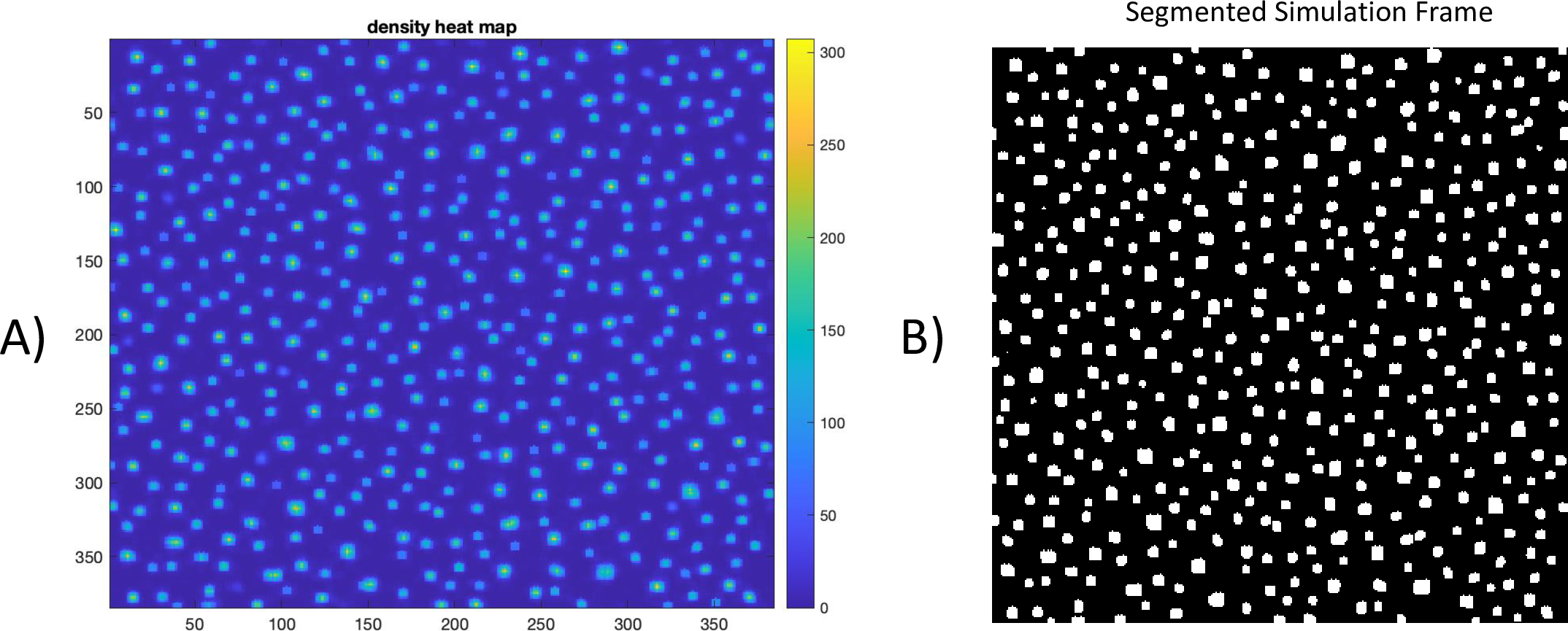

Supplement: S19 Fig — A) Density heat map of simulation results. For every pixel, the number of agents within a radius of 3 pixels is used to calculate the local cell density. B) Segmentation results after only considering pixels with a density value greater than 40 and aggregates with an area greater than 10 pixels. (TIF) [file pcbi.1009153.s031.tif]
